# Peripheral Inhibitor of AChE, Neostigmine, Prevents the Inflammatory Dependent Suppression of GnRH/LH Secretion during the Follicular Phase of the Estrous Cycle

**DOI:** 10.1155/2017/6823209

**Published:** 2017-08-15

**Authors:** Andrzej P. Herman, Janina Skipor, Agata Krawczyńska, Joanna Bochenek, Karolina Wojtulewicz, Hanna Antushevich, Anna Herman, Kamila Paczesna, Katarzyna Romanowicz, Dorota Tomaszewska-Zaremba

**Affiliations:** ^1^The Kielanowski Institute of Animal Physiology and Nutrition, Polish Academy of Sciences, Jabłonna, Poland; ^2^Institute of Animal Reproduction and Food Research, Polish Academy of Sciences, Olsztyn, Poland; ^3^Faculty of Cosmetology, The Academy of Cosmetics and Health Care, Warsaw, Poland

## Abstract

The study was designed to test the hypothesis that the inhibition of acetylcholinesterase (AChE) activity at the periphery by Neostigmine (0.5 mg/animal) will be sufficient to prevent inflammatory dependent suppression of the gonadotropin-releasing hormone (GnRH)/luteinising hormone (LH) secretion in ewes in the follicular phase of the estrous cycle, and this effect will be comparable with the systemic AChE inhibitor, Donepezil (2.5 mg/animal). An immune/inflammatory challenge was induced by peripheral administration of lipopolysaccharide (LPS; 400 ng/kg). Peripheral treatment with Donepezil and Neostigmine prevented the LPS-induced decrease (*P* < 0.05) in LH*β* gene expression in the anterior pituitary gland (AP) and in LH release. Moreover, Donepezil completely abolished (*P* < 0.05) the suppressory effect of inflammation on GnRH synthesis in the preoptic area, when pretreatment with Neostigmine reduced (*P* < 0.05) the decrease in GnRH content in this hypothalamic structure. Moreover, administration of both AChE inhibitors diminished (*P* < 0.05) the inhibitory effect of LPS treatment on the expression of GnRH receptor in the AP. Our study shows that inflammatory dependent changes in the GnRH/LH secretion may be eliminated or reduced by AChE inhibitors suppressing inflammatory reaction only at the periphery such as Neostigmine, without the need for interfering in the central nervous system.

## 1. Introduction

An immune/inflammatory challenges caused by the bacterial or viral infection could be one of the reasons of reproductive disorders in both humans and animals [1]. It is postulated that the interaction between the immune and neuroendocrine systems may occur at all levels of the neurohormonal system of hypothalamic-pituitary-gonadal (HPG) axis controlling the female reproductive process. A particularly important role in the communication between these two systems is played by the hypothalamus, the part of the brain responsible for the integration and processing of signals from the nervous, endocrine, and immune systems, what is essential for maintaining the homeostasis. The hypothalamus plays a key role in the control of reproduction in females by tonic release of gonadotropin-releasing hormone (GnRH) to the hypothalamic-pituitary portal circulation. In turn, GnRH regulates the secretion of luteinising hormone (LH) and follicle-stimulating hormone (FSH) from the gonadotropic cells in the anterior pituitary gland (AP) [2].

It was previously reported that both acute and prolonged inflammation induced by peripheral administration of bacterial endotoxin-lipopolysaccharide (LPS) may disturb the secretion of GnRH and LH [3, 4]. The study on ewes in the follicular phase of the estrous cycle showed that inflammation interrupted the preovulatory estradiol increase and delayed or blocks the subsequent LH and FSH surges [5]. This suppressive effect of inflammation on the gonadotropins secretion seems to be mediated via proinflammatory cytokines reaching the hypothalamic area during immune challenges [6]. Interleukin- (IL-) 1*β* and tumor necrosis factor *α* (TNF*α*) may represent the major proinflammatory cytokines mediating the LPS-induced suppression of GnRH and LH release, whereas the role of IL-6 in this process seems to be marginal [6–8].

One of the endogenous mechanisms involved in the regulation of immune response and cytokine secretion is the cholinergic anti-inflammatory pathway. It had been previously described that the cholinergic anti-inflammatory pathway could be activated by stimulation of the vagus nerve thereby increasing the acetylcholine (ACh) secretion [9]. This anti-inflammatory mechanism could be also activated by pharmacological blockade of the acetylcholinesterase (AChE) activity, the enzyme responsible for the degradation of ACh. In vitro studies revealed that ACh acting probably via nicotinic receptor CHRNA7 reduced LPS-stimulated release of proinflammatory cytokines, including IL-1*β*, IL-6, and TNF*α* [10]. In vivo study also showed that blockade of AChE activity reduced synthesis of IL-1*β* during peripheral inflammation in mouse [11] and sheep [12] hypothalamus. Moreover, our previous study on ewes showed that the activation of the cholinergic anti-inflammatory pathway by Rivastigmine may abolish the inhibitory effect of LPS administration on the GnRH/LH secretion and reduced the release of stress markers such as cortisol and prolactin [13]. However, Rivastigmine, AChE inhibitor used in this study, exhibits the systemic action; therefore, it blocks the AChE activity both in the brain parenchyma and in the periphery, because it easily crosses the blood-brain barrier (BBB). Therefore, it could not be concluded whether and to what extent the observed reduction of IL-1*β* synthesis in the central nervous system (CNS) and changes in hormone secretion resulted from the inhibition of the AChE activity in the CNS or the reduction in peripheral levels of proinflammatory cytokines. The results of experiments performed on mice suggest that only the reduction of circulating concentration of proinflammatory cytokines under certain conditions may be sufficient to significant inhibition of LPS-induced synthesis of IL-1*β* in the CNS [11]. This study suggests that, to disturb the functioning of CNS, the blood level of immune mediators has to enrich a critical level. Therefore, the reduction of proinflammatory cytokine concentration below this critical value may block the transmission of the inflammatory signal into the brain parenchyma. These all suggest that the activation of the cholinergic anti-inflammatory pathway only in the periphery may be sufficient to stop excessive increase in the concentration of proinflammatory cytokines in the blood, which in turn may be sufficient to reverse the negative effects of immune stress on the GnRH/LH, without providing the AChE inhibitor and direct interference in the CNS. Therefore, in the present study we used two AChE inhibitors differing in the ability to cross the BBB: Donepezil which greatly cross the BBB and Neostigmine which does not penetrate the BBB.

The present study tested the hypothesis that the inhibition of AChE activity at the periphery by Neostigmine will be sufficient to prevent the LPS-induced suppression of GnRH/LH secretion in ewes in the follicular phase of the estrous cycle, and this effect will be comparable with the systemic action of Donepezil.

## 2. Materials and Methods

### 2.1. Animals

The studies were performed on adult, 2-year-old Blackhead ewes during the reproductive season (September-October). The ewes were maintained in good conditions; that is, their body condition was estimated at 3 in a five-point scale [14] and the animals were acclimated to the experimental conditions for one month. The ewes had constant visual contact with each other in order to avoid isolation stress. The animals were fed a constant diet of commercial concentrates with hay and water available ad libitum, according to the recommendations proposed by the National Research Institute of Animal Production for adult ewes [15].

In order to best standardize experimental conditions the stage of the estrous cycle of ewes were synchronized by the Chronogest® CR (Merck Animal Health, Boxmeer, Netherlands) method using an intravaginal sponge impregnated with 20 mg of a synthetic progesterone-like hormone. All ewe had Chronogest CR sponges placement for 14 days. Following sponge removal, the ewes will receive an intramuscular injection of 500 iu pregnant mare's serum gonadotropin (PMSG) (Merck Animal Health, Boxmeer, the Netherlands). The experimental procedure was performed 24 h following PMSG injection. In treated animals, the immune stress was induced by the intravenous (iv.) injection of LPS from* Escherichia coli* 055:B5 (Sigma-Aldrich, St. Louis, MO, USA) in a dose of 400 ng/kg, dissolved in saline (0.9% w/v NaCl) (Baxter, Deerfield, IL, USA) at a concentration of 10 mg/L.

All procedures were performed with agreement of the Local Ethics Committee of Warsaw University of Life Sciences-SGGW.

### 2.2. Experimental Procedures

Venous catheters were implanted into the jugular vein on the day prior to the experiment. Ewes (*n* = 36) were randomly divided into six experimental groups (Table 1). Jugular blood samples (6 ml) were taken for measurement of the peripheral hormone at 15 min intervals beginning 2 h before the iv. administration of LPS or an equivalent volume of saline injection and continuing for 3 h. Half hour prior to LPS/saline treatment the animals were slowly intravenously treated with saline (groups 1 and 2) or suitable AChE inhibitor (groups 3, 4, 5, and 6) (Table 1). After the blood collection, the animals were immediately euthanized (3 h after LPS or saline administration) and the brains were rapidly removed from the skulls. From the ovine brains four hypothalamic structures were dissected due to their involvement in the GnRH-ergic activity. In the hypothalamus of sheep GnRH-ergic neurons did not form dense clusters, but they spread from brain septum and the horizontal diagonal band of Broca, through the preoptic area (POA), anterior hypothalamus (AHA), and medial basal hypothalamus (MBH) [2]. However, most of GnRH neurons have their pericarions located in the POA; therefore, it plays a pivotal role in GnRH synthesis. The majority of GnRH-ergic neurons send their axonal projection to the median eminence (ME) where GnRH is released to the hypophyseal portal system [2]. The hypothalamic structures such as the POA, AHA, MBH, and ME were dissected according to stereotaxic atlas of the sheep brain [18] as it was described elsewhere [13]. Landmarks were the mammillary body, median eminence, and optic chiasm. The depths of the cuts were 2 to 2.5 mm for MBH and 2.5 to 3 mm for AHA and POA. All tissues were frozen immediately after collection in liquid nitrogen and then will be stored at −80°C.

### 2.3. Assays

#### 2.3.1. Radioimmunoassay for LH

The plasma LH concentration was assayed with a double-antibody RIA using anti-ovine-LH and anti-rabbit-*γ*-globulin antisera and ovine standard (teri.oLH, Tucker Endocrine Research Institute), according to Stupnicki and Madej [19]. The assay sensitivity was 0.3 ng/ml and the intra- and interassay coefficients of variation were 8% and 11.5%, respectively.

#### 2.3.2. Radioimmunoassay for FSH

The concentration of FSH was determined by double-antibody radioimmunoassay (RIA) using anti-ovine-FSH (teri.anti-oFSH) and anti-rabbit-*γ*-globulin antisera, according to L'Hermite et al. [20]. The anti-FSH, as well as the FSH standard (teri. oFSH-and teri. FSH ig), was kindly supplied by Dr. Reichert Jr. (Tucker Endocrine Research Institute LLC, Atlanta, Georgia, USA). The assay sensitivity was 1.5 ng/ml and the intra- and interassay coefficients of variation were 3.5% and 11.3%, respectively.

#### 2.3.3. Radioimmunoassay for Prolactin

The plasma prolactin concentration was assayed by a radioimmunoassay double-antibody method, using specific antiovine prolactin and anti-rabbit-*γ*-globulin antisera according to Wolińska et al. [21]. The prolactin standard for iodination was obtained according to the method described by H. Kochman and K. Kochman [22]. The assay sensitivity for prolactin was 2 ng/ml, and the intra- and interassay coefficients of variation were 9% and 12%, respectively.

#### 2.3.4. Radioimmunoassay for Cortisol

The cortisol concentrations were determined by radioimmunoassay (RIA) according to Kokot and Stupnicki [23], using rabbit anticortisol antisera (R/75) and an HPLC-grade cortisol standard (Sigma-Aldrich, St. Louis, MO, USA). The assay sensitivity was 1 ng/ml and the intra- and interassay coefficients of variation for cortisol were 9% and 12%, respectively.

#### 2.3.5. ELISA Assay for the GnRH Concentration in the POA

The concentrations of GnRH in the POA were determined with a commercial GnRH ELISA kit (BlueGene Biotech Co., Ltd., China) dedicated for sheep. All stages of GnRH analysis were performed according manufacturer's protocol. The tissues were homogenized in 400 *μ*l of phosphate buffered saline (0.02 M). Then homogenates were subjected to two freeze-thaw cycles to further break the cell membranes. After that, the homogenates were centrifugated for 15 min at 1500 ×g in 4°C. The supernatants were aliquoted and stored until assay in −80°C. All steps in the assays were performed according to the manufacturer's instructions. The incubation of plates and absorbance measurement at 450 nm were performed using a VersaMax reader (Molecular Devices LLC, Sunnyvale, California, United States). The assay sensitivity was 1.0 pg/ml. The values of GnRH concentration were normalised to total protein content in each sample assayed using Bradford method.

#### 2.3.6. Determining the Relative Gene Expression

Total RNA from the hypothalamic structure and AP were isolated using the components of NucleoSpin® RNA/Protein Kit (MACHEREY-NAGEL Gmbh & Co., Düren, Germany) according to a manufacturer's instruction. The purity and concentration of isolated RNA were spectrophotometrically quantified by measuring the optical density at 230, 260, and 280 nm in a NanoDrop 1000 instrument (Thermo Fisher Scientific Inc., Waltham, USA). The RNA integrity was verified by electrophoresis using 1% agarose gel stained with ethidium bromide (Sigma-Aldrich, St. Louis, MO, USA). Maxima™ First Strand cDNA Synthesis Kit for RT-qPCR (Thermo Fisher Scientific Inc., Waltham, USA) was used to prepare cDNA synthesis. As a starting material for this PCR synthesis 2 *μ*g of total RNA was used.

Real-time RT-PCR was carried out using HOT FIREPol EvaGreen® qPCR Mix Plus (Solis BioDyne, Tartu, Estonia) components and HPLC-grade oligonucleotide primers synthesised by Genomed (Poland), according to the method described elsewhere [16]. Specific primers for determining the expression of housekeeping genes and the genes of interest were chosen based on our previous studies (Table 2). One tube contained 4 *μ*l PCR Master Mix (5x), 14 *μ*l RNase-free water, 1 *μ*l primers (0.5 *μ*l each, working concentration was 0.25 *μ*M), and 1 *μ*l cDNA template. The tubes were run on the Rotor Gene 6000 (Qiagen, Duesseldorf, Germany). The following protocol was used: 95°C in 15 min for activating Hot Star DNA polymerase and finally the PCR including 30 cycles at 95°C in 10 sec for denaturation, 60°C in 20 sec for annealing, and 72°C in 10 sec for extension. After the cycles, a final melting curve analysis under continuous fluorescence measurements was performed to confirm the specificity of the amplification.

Relative gene expression was calculated using the comparative quantification option [24] of the Rotor Gene 6000 software version 1.7 (Qiagen, Dusseldorf, Germany). Three housekeeping genes were examined: glyceraldehyde-3-phosphate dehydrogenase (GAPDH), *β*-actin (ACTB), and histone deacetylase 1 (HDAC1). The mean expression of these three housekeeping genes was used to normalise the expression of the analysed genes. The results are presented in arbitrary units, as the ratio of the target gene expression to the mean expression of the housekeeping genes.

#### 2.3.7. Western Blot Assays for GnRHR Expression in the AP

Before electrophoresis, the protein concentrations of samples isolated previously from the AP using the NucleoSpin RNA/Protein Kit (MACHEREY-NAGEL Gmbh & Co., Düren, Germany) were quantified using a Protein Quantification Assay Kit (MACHEREY-NAGEL Gmbh & Co., Düren, Germany). The appropriate volume of molecular grade water (Sigma-Aldrich, St. Louis, MO, USA) was added to a volume of sample containing 50 *μ*g of total protein to bring the total sample volume to 20 *μ*l. Next, 19 *μ*l of Laemmli buffer (Sigma-Aldrich, St. Louis, MO, USA) and 1 *μ*l of *β*–mercaptoethanol (Sigma-Aldrich, St. Louis, MO, USA) were added. Such mixtures were boiled for 3 min. Electrophoresis was then performed in the presence of molecular weight markers (Spectra Multicolor Broad Range Protein Ladder, Thermo Fisher Scientific Inc., Waltham, MA, USA). Denatured samples and molecular weight standards were loaded onto 4–12% polyacrylamide gels and subjected to electrophoresis in a Tris-glycine running buffer using the Protean II xi Cell (Bio-Rad Laboratories, Inc., Hercules, CA, USA) according to the manufacturer's instructions. Next, proteins were transferred in Tris-glycine blotting buffer to polyvinylidene difluoride membranes (Immobilon™-P (0.45 *μ*m), Merck KGaA, Darmstadt, Germany) using the Trans-Blot® SD Semi-Dry Transfer Cell (Bio-Rad Laboratories, Inc., Hercules, CA, USA) for 30 min at 20 V. The membranes were blocked for 1 h at room temperature in blocking buffer made up of Tris buffered saline at pH 7.5 with 0.05% Tween-20 (TBST) (Sigma-Aldrich, St. Louis, MO, USA) containing 3% bovine serum albumin fraction V (Sigma-Aldrich, St. Louis, MO, USA). Next, membranes were incubated overnight at 4°C with the following primary antibodies: goat anti-GnRHR polyclonal antibody (cat. number sc-8682, Santa Cruz Biotechnology Inc., Dallas, USA) and mouse anti-ACTB monoclonal antibody (cat. number sc-47778, Santa Cruz Biotechnology Inc., Dallas, USA) dissolved in blocking buffer at dilutions of 1 : 500 and 1 : 1000, respectively. After washing three times, membranes were incubated with the following secondary HRP conjugated antibodies: donkey anti-goat IgG-HRP (cat. number sc-2304, Santa Cruz Biotechnology Inc., Dallas, TX, USA) and goat anti-mouse IgG1 heavy chain (HRP) (cat. number ab97240, Abcam, Cambridge, UK) dissolved in blocking buffer at a dilution of 1 : 10,000. After washing three times, the membranes were visualised using chromogenic detection with a Pierce 1-step TMB-blotting substrate solution (Thermo Fisher Scientific, Waltham, MA, USA). After visualisation, the membranes were dried and scanned using an Epson Perfection V370 Photo scanner (Seiko Epson Corporation, Suwa, Japan). Densitometric analysis of the scanned membrane was performed using the software ImageJ (Research Services Branch, National Institute of Mental Health, Bethesda, MD, USA).

### 2.4. Statistical Analysis of Data

The results of hormones concentration are presented as the mean ± SEM. All experiments consisted of a baseline period when no treatment was given (2 to 0.5 h before) and a period after treatment (1 to 3 h after). To identify treatment effects, the mean values for the baseline and treatment periods were obtained. To compare the baseline period when no treatment was given and a period after treatment, the obtained data were compared with use of Student's *t*-test for dependent samples (“repeated measures”). Statistical significance was defined as *P* < 0.05.

The results of blood hormones concentration obtained only after treatment period, GnRH content in the POA, GnRHR protein expression, and all examined genes expression were analysed using a two-way ANOVA, the examined factors were inflammatory state and AChE inhibitor treatment (Donepezil or Neostigmine). Before ANOVA was conducted its two assumptions were checked: normality (Shapiro-Wilk's test) and homogeneity of the variances (Levene's test). When a significant treatment by time interaction was observed, a post hoc analysis was conducted to identify treatment effects. Fisher's least significant difference post hoc test was used to compare precompared with posttreatment values. Statistical significance was defined as *P* < 0.05.

The statistical analysis was performed using the STATISTICA 10 software (StatSoft Inc., Tulsa, OK, USA).

## 3. Results

### 3.1. Effect of AChE Inhibitors and LPS Injection on LH, FSH, Prolatin, and Cortisol Release

**Figure 1:**
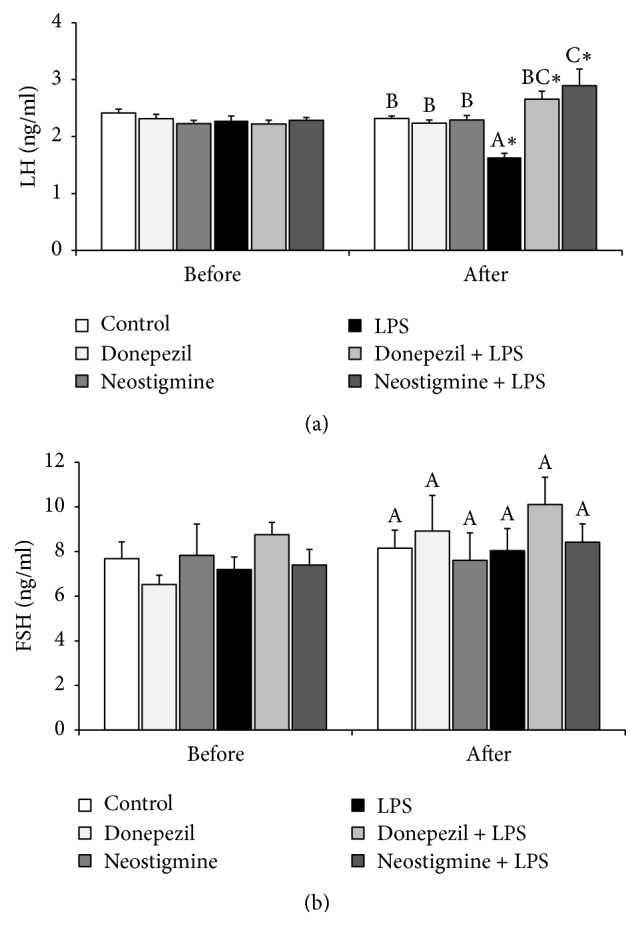
Effect of lipopolysaccharide (LPS; 400 ng/kg; iv.) and acetylcholinesterase inhibitors: Donepezil (2.5 mg/animal; iv.) and Neostigmine (0.5 mg/animal; iv.) injections on blood concentration of luteinising hormone (LH) (a) and follicle-stimulating hormone (FSH) (b) concentration in the blood plasma. The data are presented as the mean value ± SEM. All experiments consisted of a baseline period when no treatment was given (2 to 0.5 h before) and a period after treatment (1 to 3 h after). *∗*: asterisk indicates statistically significant differences between the period when no treatment was given and a period after treatment according to Student's* t*-test for dependent samples (“repeated measures”). The results of blood hormones concentration obtained only after treatment period were analysed using a two-way ANOVA. Different capital letters indicate significant differences according to a two-way ANOVA followed by Fisher's post hoc test. Statistical significance was defined as *P* < 0.05.

**Figure 2:**
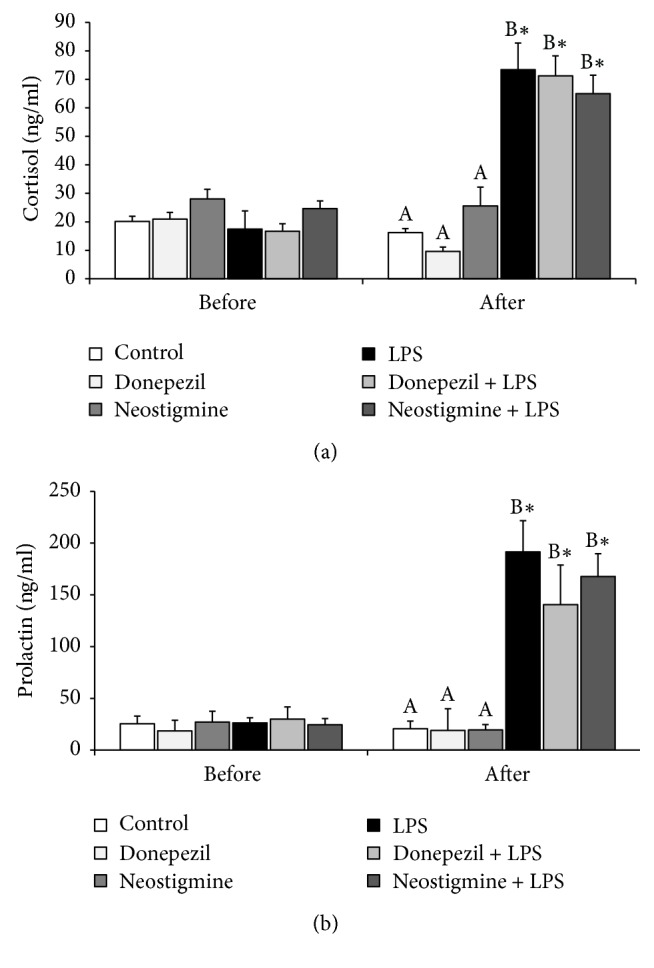
Effect of lipopolysaccharide (LPS; 400 ng/kg; iv.) and acetylcholinesterase inhibitors: Donepezil (2.5 mg/animal; iv.) and Neostigmine (0.5 mg/animal; iv.) injections on blood concentration of stress markers: cortisol (a) and prolactin (b) concentration in the blood plasma. The data are presented as the mean value ± SEM. All experiments consisted of a baseline period when no treatment was given (2 to 0.5 h before) and a period after treatment (1 to 3 h after). *∗*: asterisk indicates statistically significant differences between the period when no treatment was given and a period after treatment according to Student's *t*-test for dependent samples (“repeated measures”). The results of blood hormones concentration obtained only after treatment period were analysed using a two-way ANOVA. Different capital letters indicate significant differences according to a two-way ANOVA followed by Fisher's post hoc test. Statistical significance was defined as *P* < 0.05.

Both Donepezil and Neostigmine treatment prevented the LPS-induced decrease (*P* < 0.05) in plasma concentrations of LH. Moreover, in animals treated with Neostigmine and LPS the plasma concentration of LH was higher (*P* < 0.05) than in the control group (Figure 1(a)). In contrast, the peripheral concentrations of FSH were unaffected by all treatments (Figure 1(b)). Endotoxin injection increased (*P* < 0.05) the concentration of stress markers: cortisol (Figure 2(a)) and prolactin (Figure 2(b)), and these increases were not influenced by the AChE inhibitors treatment.

### 3.2. Effect of AChE Inhibitors and LPS Injection on GnRH Content in the POA

**Figure 3:**
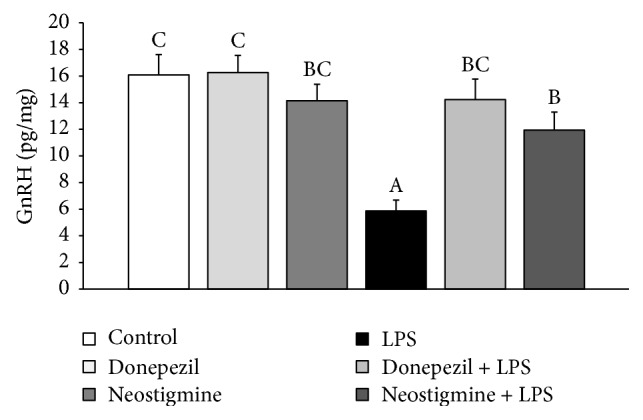
Effect of lipopolysaccharide (LPS; 400 ng/kg; iv.) and acetylcholinesterase inhibitors: Donepezil (2.5 mg/animal; iv.) and Neostigmine (0.5 mg/animal; iv.) injections on blood the content of gonadotropin-releasing hormone (GnRH) in the hypothalamus of ewes during the follicular phase of the estrous cycle. The data are presented as the mean value ± SEM. The results were analysed using a two-way ANOVA. Different capital letters indicate significant differences according to a two-way ANOVA followed by Fisher's post hoc test. Statistical significance was defined as *P* < 0.05.

Endotoxin treatment decreased (*P* < 0.05) the content of GnRH in the POA. Preceding injection of Donepezil completely abolished this suppressory effect of inflammation on the GnRH content in the POA, when pretreatment with Neostigmine reduced (*P* < 0.05) the negative effect of inflammation on the GnRH content in the POA, but it stayed significantly lower compared with the control group (Figure 3).

### 3.3. Effect of AChE Inhibitors and LPS Injection on GnRHR Protein Expression in the AP

**Figure 4:**
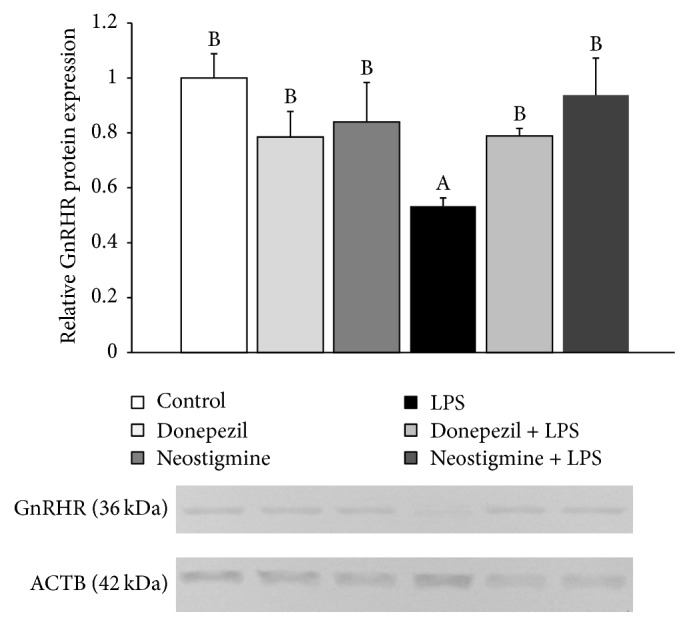
Effect of lipopolysaccharide (LPS; 400 ng/kg; iv.) and acetylcholinesterase inhibitors: Donepezil (2.5 mg/animal; iv.) and Neostigmine (0.5 mg/animal; iv.) injections on the relative protein expression (mean ± SEM; *n* = 6 animals per group) of gonadotropin-releasing hormone receptor (GnRHR) in the anterior pituitary of ewes during the follicular phase of the estrous cycle. The data are presented as the mean value ± SEM. The results were analysed using a two-way ANOVA. Different capital letters indicate significant differences according to a two-way ANOVA followed by Fisher's post hoc test. Statistical significance was defined as *P* < 0.05.

Inflammation reduced (*P* < 0.05) expression of GnRHR in the AP of ewes but the preceding injection of Donepezil and Neostigmine abolished the inhibitory effect of LPS treatment on the expression of this receptor (Figure 4).

### 3.4. Effect of AChE Inhibitors and LPS Injection on the Gene Expression in the Hypothalamus and AP

Endotoxin treatment decreased (*P* < 0.05) the level of GnRH mRNA only in the ME, but preceding injection of both AChE inhibitors prevented this effect of inflammation. It is worth mentioning that the gene expression of GnRH was not affected by any treatment in other hypothalamic structures analysed (Table 3).

In the AP, the inflammation decreased the gene expression of GnRHR and pretreatment with both AChE inhibitors did not influence the effect of inflammation on this receptor gene expression. On the other hand, preceding injection of Donepezil and Neostigmine diminish (*P* < 0.05) suppressory effect of inflammation on the LH*β* mRNA expression in the AP. No effect of any treatment on the gene expression of FSH*β* was determined. It was also determined that LPS injection stimulated (*P* < 0.05) gene expression of prolactin in the AP, and neither Donepezil nor Neostigmine influenced the level of prolactin mRNA (Table 4).

## 4. Discussion

The present study showed that peripheral AChE inhibitor, Neostigmine, the same as Donepezil, suppressed inhibitory effect of acute inflammation on LH release and LH*β* gene expression in the AP in ewes during the follicular phase of the estrous cycle. Moreover, in animals treated together with Neostigmine and LPS the circulating level of LH was higher than in the control group. The study supports the results of our previous experiment on ewes which showed that systemic AChE inhibitor successfully reduced negative effect of inflammation on LH secretion in the follicular phase ewes [13]. On the other hand, no effect of either AChE inhibitors or acute immune stress was found upon the circulating concentration of FSH. This also supports the results of previous studies indicating that acute inflammation did not influence the FSH release in both anoestrous [25] and follicular phase ewes [13]. However, other studies on ewes showed that the potency of LPS to affect the secretion of FSH may be dependent upon the duration of the inflammatory stimuli, because prolonged exposition of ewe on the action of bacterial endotoxin was found to disturb FSH release [3, 5]. It is worth mentioning that, except duration, the effect of endotoxin on gonadotropins secretion could be also dependent on the circulating concentration of ovarian steroids. It was found that endotoxin delayed the time to an experimentally induced LH surge in ovariectomized ewes but did not alter surge amplitude, duration, or incidence. This effect of LPS on the LH surge was dependent upon the moment when endotoxin was introduced relative to the onset of the estradiol signal. When endotoxin was administered early in the initial period of estrogen sensitivity, it blocked the LH surge in most ewes, but when endotoxin was administered after the period of estrogen sensitivity, the level of LH remained unaffected [26].

The changes in the endocrine activity of the AP seem to be a repercussion of events occurring at the level of hypothalamus. The study showed that preceding administration of AChE inhibitors reduced the suppressory action of acute inflammation on GnRH synthesis in the POA. However, our results suggest that in the follicular phase of the estrous cycle inflammation suppresses the GnRH synthesis at the posttranscriptional level, because no effect of LPS treatment on the gene expression of GnRH was found in the hypothalamic structures containing pericarya of GnRH neurons. This observation in the follicular phase ewes is generally consistent with the characteristic of GnRH mRNA synthesis. The previous study showed that the ratio of amount of GnRH nuclear mRNA to GnRH cytoplasmic mRNA is 1 : 2.5 and 1 : 1.5, respectively, depending on the study [27, 28]. Therefore, a greater amount of nuclear transcript provides a steady flow of GnRH mRNA to the cytoplasm, and it is generally postulated that changes in the amount of GnRH mRNA in the perikaryons are rather dependent on this mRNA turnover, both rapid accumulation and fast degradation. It should be noted that the effect of inflammation on the GnRH mRNA expression in the hypothalamus of sheep may be influenced upon the circulating concentrations of estradiol. The study on ewes during anestrous season, when the level of estradiol is presumably low, showed that endotoxin-induced inflammation decreased the transcription of GnRH mRNA in the POA [29]. Moreover, previous study showed that central action of potent proinflammatory cytokine, IL-1*β*, may be responsible for the suppression of the translational efficiency of GnRH mRNA in the rat [30] and sheep [8] hypothalamus. Therefore, it seems that in the present study the decrease found in the content of GnRH in the POA in LPS treated ewes may result from reduced translation of this decapeptide, and in turn the ability of AChE inhibitors to blockade this negative effect on the GnRH synthesis may result from the suppression of the level of central cytokines.

In the present study both Neostigmine and Donepezil treatment completely abolished LPS-induced decrease in the content of GnRH mRNA in the ME, where GnRH nerve terminals are located. This observation supports the results of previous studies, which showed that immune stress may reduce the GnRH mRNA transport to the nerve terminals, thus reducing the amount of GnRH mRNA in the ME [29], but this effect may be restrained by Rivastigmine [13]. Because it is supposed that the storage of the GnRH mRNA in the nerves terminals may be an element of the system supporting the secretion of this decapeptide, the ability of AChE inhibitors to restore the amounts of GnRH mRNA in the ME could have a profound positive influence on the GnRH secretion. This may be one of the mechanisms responsible for the protective action of the AChE inhibitors on the GnRH/LH secretion during an immune/inflammatory challenge. It was described that inflammation disturbs GnRH release in ovariectomized ewes decreasing GnRH pulse amplitude without affecting the GnRH pulse frequency [31], but our study showed that Rivastigmine not only reduced the suppressory effect of inflammation on the GnRH release but also even stimulated this neurohormone secretion into the cerebrospinal fluid of ewes in the follicular phase of the estrous cycle [13].

The ability of Neostigmine and Donepezil to block the effect of inflammation on GnRH secretion in the hypothalamus may primarily result from attenuation the inflammatory signal from periphery to the brain parenchyma. As it was mentioned above, it is considered that the main mechanism via endotoxin-induced inflammation disturbing the GnRH secretion in the hypothalamus is the central action of inflammatory cytokines. In our previous study, it was found that peripheral administration of Rivastigmine inhibited the LPS-induced synthesis of IL-1*β* in and gene expression of IL-1 receptors in the hypothalamus of ewe [12]. However, the action of Rivastigmine as well as Donepezil is systemic; these compounds inhibit the AChE activity and lead to elevation of ACh concentration in both the peripheral and central tissues [32]. However, the effectiveness of Donepezil in the prevention of inflammatory dependent changes in the GnRH/LH secretion in the follicular phase does not surprise because obtained results are concomitant with those described in our study with the use of Rivastigmine [13]. The fact that pretreatment with Neostigmine also abolished suppressive effect of LPS treatment on the GnRH/LH secretion suggests that the inhibition of proinflammatory cytokines secretion in the peripheral tissues by Neostigmine is sufficient to block the transition the inflammatory signal into the brain parenchyma, which was previously described by Pollak et al. [11]. This shows that peripheral and systemic AChE inhibitors characterize similar effectiveness in the prevention of inflammatory dependent distribution of GnRH/LH secretion and suggests that pivotal mechanism in the pathophysiology of neuroendocrine disorders occurring during an immune challenge is the elevation of peripheral proinflammatory cytokines concentration to the level necessary to transition of the information about the ongoing peripheral inflammation into the brain.

The proceeding injection of both AChE inhibitors prevented inflammatory dependent decrease in the expression of GnRHR protein but did not significantly influence on the GnRHR gene expression. During inflammatory condition, reduced expression of GnRHR in the AP may result from decreased secretion of the hypothalamic GnRH. It was described that GnRH is one of the most potent regulators of its own receptor expression. This decapeptide activates the transcriptional activity of its own receptor gene through multiple pathways, including cAMP-, PKC-, and Ca^2+^-dependent signal transduction pathways [33]. It is worth mentioning that the effect of GnRH on the expression of its own receptor in the AP is closely dependent upon character of its action. When this neurohormone is released in the pulsatile fashion, it maintains steady-state concentrations of GnRHR mRNA and numbers of GnRH receptors in the pituitary gonadotropes. But in contrast to the effects of pulsatile GnRH, continuous infusion of GnRH leads to a desensitization of gonadotropes and reduction in the number of GnRHR [34]. However, the suppression of GnRHR gene expression during inflammation may be also caused by proinflammatory cytokines and stress because it was shown that both IL-1*β* and corticotropin-releasing hormone (CRH) suppressed GnRHR expression [30, 35, 36]. The fact that preceding injection of AChE inhibitors does not allow the reduction of GnRHR expression in the AP during an immune/inflammatory challenge may have a profound importance for the reactivity of the AP because the factor determining ability and strength of the pituitary gonadotropes response to GnRH is the amount of GnRHR [34].

In the present study, neither Donepezil nor Neostigmine treatment influenced the circulating concentration of the stress markers: cortisol and prolactin which excludes that the effect of AChE injection on the GnRH/LH secretion as well as GnRHR protein expression results from the attenuation of stress reaction induced by an immune/inflammatory challenge. The stimulatory effect of immune response on the release of cortisol and prolactin has been previously described in sheep [13, 25, 37]. Cortisol is considered as an important inhibitor of the HPG axis activity, targeting the LH release [37]. However, the suppressive effect of cortisol on the release of LH release depends on the reproductive status of ewes. The ovarian steroids, particularly estradiol, enable the cortisol suppression of LH pulse frequency in sheep. Whereas cortisol seems to minimally affect LH release in the ovariectomized ewes devoid of gonadal steroids [38, 39]. Moreover, it was found that the other components of the HPA axis such as CRH and arginine vasopressin may inhibit the pulsatile GnRH/LH secretion [40]. Also prolactin may suppress LH secretion. It is known that the physiological states associated with elevated prolactin blood concentration (i.e., pregnancy, pseudopregnancy, postpartum, and lactation); the LH secretion is always decreased [41]. Circulating prolactin crosses the blood-cerebrospinal fluid barrier and reaches the brain parenchyma; therefore, the prolactin dependent inhibition of LH release could result from its action on the GnRH secretion. The results of in vitro studies conducted on the GT1 neuronal cell line showed that prolactin directly inhibits GnRH release and possibly gene expression in these cells [41]. In our previous study Rivastigmine treatment decreased the plasma concentration of these hormones in ewes but not to the control values [13]. However, the background of this Rivastigmine action was not completely clear because ACh is considered to be a stimulant of cortisol [42] and prolactin [43] release, as well as the hypothalamus-pituitary-adrenal (HPA) axis activator [44]. It was speculated that the reduction of cortisol and prolactin release might result from the analgesic action of ACh inhibitors modulating the inflammatory pain [45] and decreasing the production of proinflammatory cytokines which are also able to stimulate the activity of the HPA axis [46]. The present study suggests that the stress-reducing effect of AChE inhibitors may be not universal property of all these compounds or may depend upon used dose of the drug. However, obtained results support the current view about no pivotal role of cortisol in the inhibition of the HPG axis during inflammatory conditions. This observation is generally consistent with the study which showed that the activation of the HPA axis is not essential for reproductive disorders during endotoxin-induced inflammatory challenges [37].

From one hand, it seems that our present study also negatively tested our hypothesis formulated in the study with the usage of Rivastigmine claiming that stimulating effect of the AChE inhibitor on the GnRH secretion during immune stress may result from the accumulation of ACh in the brain. This thesis was justified because the regions of the brain that exhibit cholinergic activity have projections to the POA and therefore may regulate GnRH neuron activity [47, 48]. Moreover, in vitro experiment performed on rat hypothalamic tissue cultures demonstrated that ACh stimulated GnRH release [49]. An in vitro study performed on rat hypothalamic neurons and GT1-7 line cells showed that ACh modulated GnRH release in an enhanced way and acted through different cholinergic receptor subtypes to exert stimulatory and inhibitory effects [50]. However, in our study animals treated only with Donepezil or Neostigmine did not show any changes in the GnRH/LH secretion which suggests that maintaining of undisturbed GnRH/LH secretion in animals concomitant treated with LPS rather results from the anti-inflammatory action of this AChE inhibitors than simply from accumulation of ACh in the hypothalamus. On the other hand, the reactivity of the brain tissues during inflammatory condition may be changed. Therefore, certainly it cannot be stated that ACh does not play some role in the AChE inhibitors action on the secretion of GnRH and/or LH during inflammation because in this physiological state it may influence the responsiveness of both the hypothalamic and AP tissues on the ACh action. It was previously found that LPS influences the profile of ACh receptors which may change responsiveness of the cells on the action of this neurotransmitter [51]. In our study we determined that in animals treated with Neostigmine and LPS the mean circulating concentration of LH was higher than in the control group, but this effect was not parallel to the changes in the GnRH content in the POA. This suggests that the LH secretion is enhanced by the peripheral factors reaching directly the AP. Although the ex vivo study suggested that stimulatory action of ACh on the LH secretion from the AP is indirect and is targeted on the stimulation of the hypothalamic GnRH release [52], more present study showed that the ACh receptors are present and active in the majority of AP cells [53]. Therefore, it cannot be excluded that at least partially the stimulatory effect of Neostigmine and LPS treatment on the LH secretion may result from the accumulation of ACh in the blood which directly affects the secretory activity of AP.

In summary, our study showed that peripheral inhibitor of AChE activity, Neostigmine, effectively abolished the suppressive effect of acute inflammation on the GnRH/LH secretion and this effect was generally similar to the systemic action of Donepezil. This indicates that inflammatory dependent changes in the GnRH/LH secretion may be eliminated or reduced by the compounds suppressing inflammatory reaction only at the periphery without the need for interfering in the CNS. Our study suggests that AChE inhibitors not capable of crossing the BBB might potentially be used in the therapy of inflammatory induced neuroendocrine disorders.

## Figures and Tables

**Table 1:** The scheme of the experiment.

Group	Number of animals	Experimental treatment I (iv.)	Dose [mg/animal]	Experimental treatment II (iv.)	Dose [ng/kg]
1: control	6	NaCl	0	NaCl	0
2: LPS treated	6	NaCl	0	LPS	400
3: Donepezil treated	6	Donepezil	2.5	NaCl	0
4: Neostigmine treated	6	Neostigmine	0.5	NaCl	0
5: Donepezil + LPS treated	6	Donepezil	2.5	LPS	400
6: Neostigmine + LPS treated	6	Neostigmine	0.5	LPS	400

*Total amount of animals*	*36*				

**Table 2:** All genes analyzed by real-time PCR are listed with their full name and abbreviation.

GenBank acc. number	Gene	Amplicon size [bp]	Forward/reverse	Sequence 5′→3′	References
NM_001034034	*GAPDH* *glyceraldehyde-3-phosphate dehydrogenase*	134	forward	AGAAGGCTGGGGCTCACT	[16]
			reverse	GGCATTGCTGACAATCTTGA	
U39357	*ACTB* *beta actin*	168	forward	CTTCCTTCCTGGGCATGG	[16]
			reverse	GGGCAGTGATCTCTTTCTGC	
BC108088.1	*HDAC1* histone deacetylase 1	115	forward	CTGGGGACCTACGGGATATT	[16]
			reverse	GACATGACCGGCTTGAAAAT	
NM_001009397	*GnRHR* *gonadotropin-releasing hormone receptor*	150	forward	TCTTTGCTGGACCACAGTTAT	[17]
			reverse	GGCAGCTGAAGGTGAAAAAG	
U02517	*GnRH* *gonadotropin-releasing hormone*	123	forward	GCCCTGGAGGAAAGAGAAAT	[17]
			reverse	GAGGAGAATGGGACTGGTGA	
X52488	*LHB* *luteinizing hormone beta-subunit*	184	forward	AGATGCTCCAGGGACTGCT	[17]
			reverse	TGCTTCATGCTGAGGCAGTA	
X15493	*FSHB* *follicle stimulating hormone beta-subunit*	131	forward	TATTGCTACACCCGGGACTT	[17]
			reverse	TACAGGGAGTCTGCATGGTG	
NM_001009306	*PRL* *prolactin*	131	forward	CCTCTCCTCGGAAATGTTCA	[17]
			reverse	AGGACTTCATGGTGGGTCTG	

**Table 3:** Effect of lipopolysaccharide (LPS; 400 ng/kg; iv.) and acetylcholinesterase inhibitors: Donepezil (2.5 mg/animal; iv.) and Neostigmine (0.5 mg/animal; iv.) injections on the relative gene expression (mean ± SEM; *n* = 6 animals per group) of gonadotropin-releasing hormone (GnRH) in the hypothalamus of ewes during the follicular phase of the estrous cycle. POA: the preoptic area; AHA: the anterior hypothalamus; MBH: the medial basal hypothalamus; ME: the median eminence; control: group injected with saline; Don.: group treated with Donepezil; Neo.: group injected with Neostigmine; LPS: group which received the endotoxin injection; Don. + LPS: group treated with both Donepezil and LPS; Neo. + LPS: group treated with both Neostigmine and LPS. In all hypothalamic structures gene expression data were normalised to the average relative level of gene expression in the control ewes, which was set to 1.0. Different capital letters indicate significant (*P* < 0.05) differences according to a two-way ANOVA followed by Fisher's post hoc test.

Structure	GnRH relative gene expression					
	Control	Don.	Neo.	LPS	Don. + LPS	Neo. + LPS
POA	1 ± 0.1^A^	0.9 ± 0.1^A^	0.9 ± 0.2^A^	0.8 ± 0.2^A^	0.9 ± 0.1^A^	1 ± 0.2^A^
AHA	1 ± 0.1^A^	1.1 ± 0.1^A^	0.8 ± 0.2^A^	0.7 ± 0.1^A^	0.9 ± 0.1^A^	0.9 ± 0.1^A^
MBH	1 ± 0.1^A^	0.8 ± 0.1^A^	0.8 ± 0.2^A^	1.1 ± 0.1^A^	1 ± 0.1^A^	1.1 ± 0.2^A^
ME	1 ± 0.2^B^	1.3 ± 0.2^B^	1 ± 0.2^B^	0.1 ± 0.1^A^	0.7 ± 0.1^B^	1.2 ± 0.2^B^

**Table 4:** Effect of lipopolysaccharide (LPS; 400 ng/kg; iv.) and acetylcholinesterase inhibitors: Donepezil (2.5 mg/animal; iv.) and Neostigmine (0.5 mg/animal; iv.) injections on the relative gene expression (mean ± SEM; *n* = 6 animals per group) of gonadotropin-releasing hormone receptor (GnRHR), luteinizing hormone *β*-subunit (LH*β*), follicle-stimulating hormone *β*-subunit (FSH*β*), and prolactin (PRL) genes in the anterior pituitary of ewes during the follicular phase of the estrous cycle. control: group injected with saline; Don.: group treated with Donepezil; Neo.: group injected with Neostigmine; LPS: group which received the endotoxin injection; Don. + LPS: group treated with both Donepezil and LPS; Neo. + LPS: group treated with both Neostigmine and LPS. The gene expression of each gene was normalised to the average relative level of gene expression in the control ewes, which was set to 1.0. Different capital letters indicate significant (*P* < 0.05) differences according to a two-way ANOVA followed by Fisher's post hoc test.

Gene	Anterior pituitary					
	Control	Don.	Neo.	LPS	Don. + LPS	Neo. + LPS
GnRHR	1 ± 0.1^BCD^	1.2 ± 0.2^BCD^	1.4 ± 0.2^D^	0.5 ± 0.1^A^	0.9 ± 0.2^ABC^	0.7 ± 0.2^AB^
LH*β*	1 ± 0.1^C^	0.9 ± 0.1^BC^	1 ± 0.1^C^	0.5 ± 0.1^A^	0.9 ± 0.1^BC^	0.8 ± 0.1^BC^
FSH*β*	1 ± 0.1^A^	0.8 ± 0.1^A^	0.8 ± 0.2^A^	1.1 ± 0.1^A^	1 ± 0.1^A^	1.1 ± 0.2^A^
PRL	1 ± 0.2^A^	1.1 ± 0.2^A^	1 ± 0.2^A^	1.6 ± 0.1^B^	1.7 ± 0.1^B^	1.7 ± 0.2^B^

## References

[1] Nepomnaschy P. A., Sheiner E., Mastorakos G., Arck P. C. (2007). Stress, immune function, and women's reproduction. *Annals of the New York Academy of Sciences*.

[2] Caldani M., Batailler M., Thiéry J. C., Dubois M. P. (1988). LHRH-immunoreactive structures in the sheep brain. *Histochemistry*.

[3] Tomaszewska-Zaremba D., Herman A., Haziak K. (2016). How does bacterial endotoxin influence gonadoliberin/gonadotropins secretion and action?. *Journal of Animal and Feed Sciences*.

[4] Herman A. P., Kopycińska K., Krawczyńska A., Romanowicz K., Tomaszewska-Zaremba D. (2014). The effect of repeated endotoxin injections on gonadotropin secretion in ewes. *Journal of Animal and Feed Sciences*.

[5] Battaglia D. F., Krasa H. B., Padmanabhan V., Viguié C., Karsch F. J. (2000). Endocrine alterations that underlie endotoxin-induced disruption of the follicular phase in ewes. *Biology of Reproduction*.

[6] Watanobe H., Hayakawa Y. (2003). Hypothalamic interleukin-1*β* and tumor necrosis factor-*α*, but not interleukin-6, mediate the endotoxin-induced suppression of the reproductive axis in rats. *Endocrinology*.

[7] Rivier C., Vale W. (1990). Cytokines act within the brain to inhibit luteinizing hormone secretion and ovulation in the rat. *Endocrinology*.

[8] Herman A. P., Misztal T., Romanowicz K., Tomaszewska-Zaremba D. (2012). Central injection of exogenous IL-1*β* in the control activities of hypothalamic-pituitary-gonadal axis in anestrous ewes. *Reproduction in Domestic Animals*.

[9] Rosas-Ballina M., Ochani M., Parrish W. R. (2008). Splenic nerve is required for cholinergic antiinflammatory pathway control of TNF in endotoxemia. *Proceedings of the National Academy of Sciences of the United States of America*.

[10] Borovikova L. V., Ivanova S., Zhang M. (2000). Vagus nerve stimulation attenuates the systemic inflammatory response to endotoxin. *Nature*.

[11] Pollak Y., Gilboa A., Ben-Menachem O., Ben-Hur T., Soreq H., Yirmiya R. (2005). Acetylcholinesterase inhibitors reduce brain and blood interleukin-1*β* production. *Annals of Neurology*.

[12] Herman A. P., Krawczyńska A., Bochenek J. (2013). Inhibition of acetylcholinesterase activity by rivastigmine decreases lipopolysaccharide-induced IL-1*β* expression in the hypothalamus of ewes. *Domestic Animal Endocrinology*.

[13] Herman A. P., Krawczyńska A., Bochenek J. (2013). The effect of rivastigmine on the LPS-induced suppression of GnRH/LH secretion during the follicular phase of the estrous cycle in ewes. *Animal Reproduction Science*.

[14] Russel A., Boden E. (1991). Body Condition Scoring of Sheep. *Sheep and Goat Practice*.

[15] Roś R. R. (1993). *Nutrient Requirements for Cattle And Sheep in The Traditional System*.

[16] Herman A. P., Krawczyńska A., Bochenek J., Antushevich H., Herman A., Tomaszewska-Zaremba D. (2014). Peripheral injection of SB203580 inhibits the inflammatory-dependent synthesis of proinflammatory cytokines in the hypothalamus. *BioMed Research International*.

[17] Herman A. P., Krawczyńska A., Bochenek J., Antushevich H., Herman A., Tomaszewska-Zaremba D. (2016). Involvement of prolactin in the meloxicam-dependent inflammatory response of the gonadotropic axis to prolonged lipopolysaccharide treatment in anoestrous ewes. *Reproduction, Fertility and Development*.

[18] Welento J., Szteyn S., Milart Z. (1969). Observations on the stereotaxic configuration of the hypothalamus nuclei in the sheep.. *Anatomischer Anzeiger*.

[19] Stupnicki R., Madej A. (1976). Radioimmunoassay of LH in blood plasma of farm animals. *Endokrinologie*.

[20] L'Hermite M., Niswender G. D., Reichert L. E., Midgley A. R. (1972). Serum follicle-stimulating hormone in sheep as measured by radioimmunoassay.. *Biology of Reproduction*.

[21] Wolińska E., Polkowska J., Domanski E. (1977). The hypothalamic centres involved in the control of production and release of prolactin in sheep. *Journal of Endocrinology*.

[22] Kochman H., Kochman K. (1977). Purification of ovine and bovine prolactins on DEAE cellulose chromatography and preparative polyacrylamide gel electrophoresis.. *Bulletin de l"Academie polonaise des sciences. Serie des sciences biologiques*.

[23] Kokot F., Stupnicki R. (1985). *Metody Radioimmunologiczne i Radiokompetyncyjne Stosowane w Klinice*.

[24] Rasmussen R., Meuer S., Wittwer C., Nakagawara K. (2001). Quantification on the LightCycler. *Rapid Cycle Real-Time PCR Methods and Applications*.

[25] Herman A. P., Romanowicz K., Tomaszewska-Zaremba D. (2010). Effect of LPS on reproductive system at the level of the pituitary of anestrous ewes. *Reproduction in Domestic Animals*.

[26] Battaglia D. F., Beaver A. B., Harris T. G., Tanhehco E., Viguié C., Karsch F. J. (1999). Endotoxin disrupts the estradiol-induced luteinizing hormone surge: interference with estradiol signal reading, not surge release. *Endocrinology*.

[27] Jakubowski M., Roberts J. L. (1994). Processing of gonadotropin-releasing hormone gene transcripts in the rat brain. *Journal of Biological Chemistry*.

[28] Yeo T. T. S., Gore A. C., Jakubowski M., Dong K. W., Blum M., Roberts J. L. (1996). Characterization of gonadotropin-releasing hormone gene transcripts in a mouse hypothalamic neuronal GT1 cell line. *Molecular Brain Research*.

[29] Herman A. P., Tomaszewska-Zaremba D. (2010). Effect of endotoxin on the expression of GnRH and GnRHR genes in the hypothalamus and anterior pituitary gland of anestrous ewes. *Animal Reproduction Science*.

[30] Kang S. S., Kim S. R., Leonhardt S., Jarry H., Wuttke W., Kim K. (2000). Effect of interleukin-1*β* on gonadotropin-releasing hormone (GnRH) and GnRH receptor gene expression in castrated male rats. *Journal of Neuroendocrinology*.

[31] Battaglia D. F., Bowen J. M., Krasa H. B., Thrun L. A., Viguié C., Karsch F. J. (1997). Endotoxin inhibits the reproductive neuroendocrine axis while stimulating adrenal steroids: a simultaneous view from hypophyseal portal and peripheral blood. *Endocrinology*.

[32] Pohanka M. (2014). Inhibitors of acetylcholinesterase and butyrylcholinesterase meet immunity. *International Journal of Molecular Sciences*.

[33] Lin X., Conn P. M. (1999). Transcriptional activation of gonadotropin-releasing hormone (GnRH) receptor gene by GnRH: involvement of multiple signal transduction pathways. *Endocrinology*.

[34] Turzillo A. M., Nolan T. E., Nett T. M. (1998). Regulation of gonadotropin-releasing hormone (GnRH) receptor gene expression in sheep: Interaction of GnRH and estradiol. *Endocrinology*.

[35] Herman A. P., Krawczyńska A., Bochenek J., Dobek E., Tomaszewska-Zaremba D. (2013). LPS-induced inflammation potentiates the IL-1*β* -mediated reduction of LH secretion from the anterior pituitary explants. *Clinical and Developmental Immunology*.

[36] Ciechanowska M., Łapot M., Malewski T., Mateusiak K., Misztal T., Przekop F. (2011). Effects of corticotropin-releasing hormone and its antagonist on the gene expression of gonadotrophin-releasing hormone (GnRH) and GnRH receptor in the hypothalamus and anterior pituitary gland of follicular phase ewes. *Reproduction, Fertility and Development*.

[37] Debus N., Breen K. M., Barrell G. K. (2002). Does cortisol mediate endotoxin-induced inhibition of pulsatile luteinizing hormone and gonadotropin-releasing hormone secretion?. *Endocrinology*.

[38] Oakley A. E., Breen K. M., Clarke I. J., Karsch F. J., Wagenmaker E. R., Tilbrook A. J. (2009). Cortisol reduces gonadotropin-releasing hormone pulse frequency in follicular phase ewes: influence of ovarian steroids. *Endocrinology*.

[39] Oakley A. E., Breen K. M., Tilbrook A. J., Wagenmaker E. R., Karsch F. J. (2009). Role of estradiol in cortisol-induced reduction of luteinizing hormone pulse frequency. *Endocrinology*.

[40] Battaglia D. F., Brown M. E., Krasa H. B., Thrun L. A., Viguié C., Karsch F. J. (1998). Systemic challenge with endotoxin stimulates corticotropin-releasing hormone and arginine vasopressin secretion into hypophyseal portal blood: coincidence with gonadotropin-releasing hormone suppression. *Endocrinology*.

[41] Milenkovió L., D'Angelo G., Kelly P. A., Weiner R. I. (1994). Inhibition of gonadotropin hormone-releasing hormone release by prolactin from GT1 neuronal cell lines through prolactin receptors. *Proceedings of the National Academy of Sciences of the United States of America*.

[42] Clyne C. D., Walker S. W., Nicol M. R., Williams B. C. (1994). The M3 muscarinic receptor mediates acetylcholine-induced cortisol secretion from bovine adrenocortical zona fasciculata/reticularis cells. *Biochemical Pharmacology*.

[43] Hall T. R., Harvey S., Chadwick A. (1985). Effects of putative neurotransmitters on release of prolactin from pituitary glands of the domestic fowl co-incubated with hypothalamic tissue. *General Pharmacology*.

[44] Calogero A. E., Gallucci W. T., Bemardini R., Saoulis C., Gold P. W., Chrousos G. P. (1988). Effect of cholinergic agonists and antagonists on rat hypothalamic corticotropin-releasing hormone secretion in vitro. *Neuroendocrinology*.

[45] Buerkle H., Boschin M., Marcus M. A. E., Brodner G., Wüsten R., Van Aken H. (1998). Central and peripheral analgesia mediated by the acetylcholinesterase- lnhibitor neostigmine in the rat inflamed knee joint model. *Anesthesia and Analgesia*.

[46] Dunn A. J. (2000). Cytokine activation of the HPA axis. *Annals of the New York Academy of Sciences*.

[47] CastaNeyra-Perdomo A., Pérez-Delgado M. M., Montagnese C., Coen C. W. (1992). Brainstem projections to the medial preoptic region containing the luteinizing hormone-releasing hormone perikarya in the rat. An immunohistochemical and retrograde transport study. *Neuroscience Letters*.

[48] Boehm U., Zou Z., Buck L. B. (2005). Feedback loops link odor and pheromone signaling with reproduction. *Cell*.

[49] Richardson S. B., Prasad J. A., Hollander C. S. (1982). Acetylcholine, melatonin, and potassium depolarization stimulate release of luteinizing hormone-releasing hormone from rat hypothalamus in vitro. *Proceedings of the National Academy of Sciences of the United States of America*.

[50] Krsmanovic L. Z., Mores N., Navarro C. E., Saeed S. A., Arora K. K., Catt K. J. (1998). Muscarinic regulation of intracellular signaling and neurosecretion in gonadotropin-releasing hormone neurons. *Endocrinology*.

[51] Chernyavsky A. I., Arredondo J., Skok M., Grando S. A. (2010). Auto/paracrine control of inflammatory cytokines by acetylcholine in macrophage-like U937 cells through nicotinic receptors. *International Immunopharmacology*.

[52] Fiorindo R. P., Martini L. (1975). Evidence for a cholinergic component in the neuroendocrine control of luteinizing hormone (LH) secretion. *Neuroendocrinology*.

[53] Pintér I., Moszkovszkin G., Némethy Z., Makara G. B. (1999). Muscarinic M1 and M3 receptors are present and increase intracellular calcium in adult rat anterior pituitary gland. *Brain Research Bulletin*.

